# Fungal Lactamases: Their Occurrence and Function

**DOI:** 10.3389/fmicb.2017.01775

**Published:** 2017-09-19

**Authors:** Minglu Gao, Anthony E. Glenn, Alex A. Blacutt, Scott E. Gold

**Affiliations:** ^1^Department of Plant Pathology, The University of Georgia, Athens GA, United States; ^2^Toxicology and Mycotoxin Research Unit, U.S. National Poultry Research Center, United States Department of Agriculture – Agricultural Research Service, Athens GA, United States

**Keywords:** soil, fungi, lactams, β-lactamases, *Fusarium verticillioides*

## Abstract

Fungi are absorptive feeders and thus must colonize and ramify through their substrate to survive. In so doing they are in competition, particularly in the soil, with myriad microbes. These microbes use xenobiotic compounds as offensive weapons to compete for nutrition, and fungi must be sufficiently resistant to these xenobiotics. One prominent mechanism of xenobiotic resistance is through production of corresponding degrading enzymes. As typical examples, bacterial β-lactamases are well known for their ability to degrade and consequently confer resistance to β-lactam antibiotics, a serious emerging problem in health care. We have identified many fungal genes that putatively encode proteins exhibiting a high degree of similarity to β-lactamases. However, fungal cell walls are structurally different from the bacterial peptidoglycan target of β-lactams. This raises the question, why do fungi have lactamases and what are their functions? Previously, we identified and characterized one *Fusarium verticillioides* lactamase encoding gene (FVEG_08291) that confers resistance to the benzoxazinoid phytoanticipins produced by maize, wheat, and rye. Since benzoxazinoids are γ-lactams with five-membered rings rather than the four-membered β-lactams, we refer to the predicted enzymes simply as lactamases, rather than β-lactamases. An overview of fungal genomes suggests a strong positive correlation between environmental niche complexity and the number of fungal lactamase encoding genes, with soil-borne fungi showing dramatic amplification of lactamase encoding genes compared to those fungi found in less biologically complex environments. Remarkably, *Fusarium* species frequently possess large (>40) numbers of these genes. We hypothesize that many fungal hydrolytic lactamases are responsible for the degradation of plant or microbial xenobiotic lactam compounds. Alignment of protein sequences revealed two conserved patterns resembling bacterial β-lactamases, specifically those possessing PFAM domains PF00753 or PF00144. Structural predictions of *F. verticillioides* lactamases also suggested similar catalytic mechanisms to those of their bacterial counterparts. Overall, we present the first in-depth analysis of lactamases in fungi, and discuss their potential relevance to fitness and resistance to antimicrobials in the environment.

## Introduction

The soil is one of the most complex habitats on earth due primarily to the diversity of microorganisms that inhabit it and the myriad biochemical products they secrete. Experiments utilizing metagenomic technologies estimate up to several million species of bacteria per gram in some naturally occurring soils (Gans, 2006). The majority of these species are, to date, uncultured. For fungi, less information is available. Earlier estimates suggested that there are approximately 1.5 million fungal species on the planet (Hawksworth, 1991), but more recent global estimates of six million soil fungi were suggested based on comprehensive molecular studies (Taylor et al., 2014). This microbial diversity creates a dynamic environment for microorganisms to communicate and compete for limited resources. Further, metabolic processes of microbes together with plants act as significant sources of chemical diversity, and microbes in the soil milieu are constantly and unavoidably exposed to foreign chemicals (xenobiotics). Some xenobiotics are easily tolerated and degraded, while others have inhibitory effects (Parkinson et al., 2001). Xenobiotics that are deleterious to the growth or metabolic activities of other microorganisms can be considered antibiotics, and play critical ecological roles in competitive interactions (Thomashow et al., 1997; Davelos et al., 2004; Kinkel et al., 2012). It has been posited that microorganisms and plants have adopted antibiotic production as offensive and/or defensive strategies to adjust to changing circumstances, allowing microbial colonization in the rhizosphere or persistence of plants in the environment (Lynch et al., 2004). Compared to the surrounding soil, the rhizosphere of plants can be particularly rich in nutrients (Marschner et al., 2004). This microbial oasis stimulates competitive and antagonistic relationships among would-be colonizers. For example, phenazine production by pseudomonads and trifolitoxin production from certain *Rhizobium* species correlate with soil survival and suppressive activity, demonstrating that antibiotic production can be integral to niche competition and microbial community structure (Mazzola et al., 1992; Robleto et al., 1998).

Antibiotic production by both plants and microbes is a remarkable strategy possibly adopted in response to their sessile nature and limited mobility, respectively (Grotewold, 2005; Agrawal, 2011). Heritable genetic alterations such as mutation, gene duplication/modification, and horizontal gene transfer (HGT) have expanded the antibiotic repertoires of plants and microbes (Soucy et al., 2015). A number of antibiotic families have been detected from soil or produced by soil microbes and display *in vivo* or *in vitro* antagonistic effects, such as penicillin, trichothecene, chloromycetin, actinomycin, clavacin, griseofulvin, etc. (Stallings, 1954; Kinsella et al., 2009). Yet, antibiotics can occur in nature at sub-inhibitory concentrations, and rather than inhibiting growth, the compounds elicit transcriptional responses suggestive of a form of microbial communication (Goh et al., 2002; Davies, 2006). In addition to microbial sources, compounds with antibiotic activity are also found in plants (VanEtten et al., 1994; Bozdogan and Appelbaum, 2004; González-Lamothe et al., 2009). Maackiain is a plant-derived antibiotic extracted from red clover and alfalfa. Previous work has shown that maackiain is toxic to several genera of fungal pathogens of legume and non-legume hosts (Duczek and Higgins, 1976; Delserone et al., 1992). Maize, wheat, and rye can constitutively produce benzoxazinones and benzoxazolinones, which help reduce insect damage and confer resistance to various fungal and bacterial pathogens (Couture et al., 1971; Baker and Smith, 1977; Glenn et al., 2016).

To combat antibiosis, bacteria have developed resistance mechanisms such as efflux pumps and hydrolytic enzymes. Notorious among the latter group, β-lactamases have been thoroughly studied due to the resistance they confer to the widespread clinically used β-lactam antibiotics. Parallel to the presence in bacteria, genes encoding “β-lactamases” are also abundant across different fungal families. In contrast to bacteria, almost nothing is known about the function of these genes in fungi. Previous work is limited to two studies on the hydrolytic function of lactamase (metallo-β-lactamase, MBL) encoding genes in *Fusarium verticillioides* and *Fusarium pseudograminearum* (Kettle et al., 2015b; Glenn et al., 2016). This evidence serves as a foundational paradigm for studying hydrolytic lactamases in fungi and prompts the hypothesis that, as in bacteria, many of these enzymes function in degradation and resistance to xenobiotic compounds. In this review, we will describe an initial look at the distribution of lactamase-encoding genes in fungi and speculate on their ecological roles. We will also describe current and planned approaches to decipher the roles of 46 lactamase-family genes in the *F. verticillioides* genome.

## Lactams—the Archetypical Class of Antibiotics

### Bactericidal β-Lactams

**FIGURE 1:**
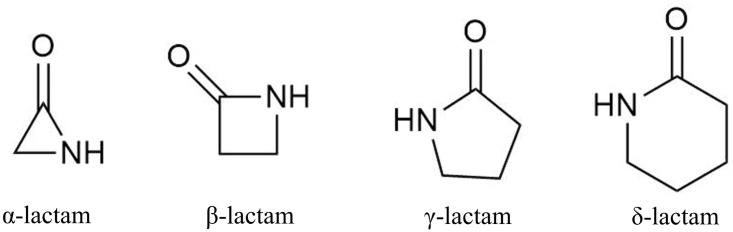
Basic lactam structures with differing ring sizes.

β-Lactams comprise the largest group of antibiotics, and they have been extensively utilized for their antibacterial effect (Tipper, 1985). Beginning with Alexander Fleming’s Nobel Prize-winning serendipitous discovery of a penicillin-producing mold, β-lactams and their semisynthetic derivatives have been the most impactful antibiotics in medicine (Demain and Elander, 1999; Lewis, 2013). Their mode of action is well characterized and involves a four-membered cyclic amide ring (**Figure 1**) that occupies the catalytic sites of transpeptidases, also referred to as penicillin-binding proteins. These proteins are essential for cross-linking peptidoglycan layers of bacterial cell walls, thus β-lactam antibiotics disrupt bacterial cell wall synthesis, resulting in cell lysis (Waxman and Strominger, 1983).

### Lactam Production in Fungi

**FIGURE 2:**
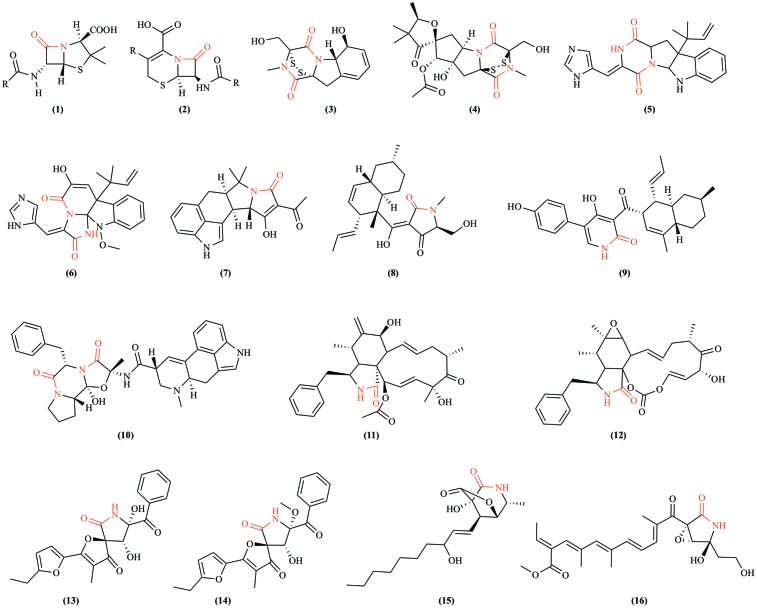
Examples of lactam-containing fungal compounds. Lactam bonds are highlighted in red. **(1)** Penicillin, the historically significant fungal lactam produced by *Penicillium chrysogenum* (Fleming, 1929); **(2)** cephalosporins, a group of bactericidal β-lactams from *Acremonium chrysogenum* (Harrison and Bratcher, 2008); **(3)** gliotoxin, a mycotoxin produced by *Aspergillus fumigatus* and several other species (Forseth et al., 2011); **(4)** sirodesmin PL, a phytotoxin produced by the fungus *Leptosphaeria maculans* causing blackleg disease of canola (Gardiner et al., 2004); **(5)** roquefortine C, a mycotoxin produced by *Penicillium* species (Kokkonen et al., 2005); **(6)** meleagrin, a bioactive alkaloid produced by deep ocean *Penicillium* (Nozawa and Nakajima, 1979); **(7)** cyclopiazonic acid, a toxic fungal secondary metabolite originally isolated from *Penicillium cyclopium* (Holzapfel, 1968); **(8)** equisetin, a *Fusarium equiseti* metabolite (Hazuda et al., 1999); **(9)** ilicicolin H is an NRPS-polyketide hybrid product discovered from *Cylindrocladium iliciola* MFC-870 and is a potent antifungal agent (Singh et al., 2011); **(10)** ergotamine, an ergopeptine and part of the ergot family of alkaloids from *Claviceps purpurea* (Schiff, 2006); **(11)** cytochalasin D, a cytostatically active metabolite isolated from *Tubercularia* species (Wang et al., 2003); **(12)** cytochalasin E from *Rhinocladiella* species (Wagenaar et al., 2000); **(13)** azaspirofuran A and **(14)** azaspirofuran B produced by *Aspergillus sydowii* (Ren et al., 2010); **(15)** awajanomycin produced by *Acremonium* species (Jang et al., 2006); **(16)** fusarin C, a mycotoxin produced by several *Fusarium* species (Wiebe and Bjeldanes, 1981).

Fungi are the original source of two foundational β-lactam antibiotics: penicillin and cephalosporin. These drugs are still industrially produced, primarily using *Penicillium chrysogenum* and *Acremonium chrysogenum* (previously *Cephalosporium*), respectively (Brakhage et al., 2009). Lactam production in fungi is frequently coordinated through the activity of gene clusters containing necessary biosynthetic enzymes and pathway-specific transcriptional regulators (Brakhage et al., 2009; Khaldi et al., 2010; Osbourn, 2010; Brakhage and Schroeckh, 2011). Fungal gene clusters are hypothesized to assist in retention of biochemical functions by reducing gene loss due to recombination in highly dynamic genomes (Osbourn, 2010). Fungal genomes provide enormous potential to produce many complex lactam-containing compounds (**Figure 2**), including higher order lactam compounds (e.g., five-membered, γ-lactam rings). Two new hetero-spirocyclic γ-lactams, azaspirofurans A and B, were isolated from a marine sediment-derived fungus *Aspergillus sydowii* (Ren et al., 2010). A maize seed-borne endophyte *Sarocladium zeae* (formerly *Acremonium zeae*) was found to produce γ-lactam compounds, named pyrrocidine A and B (He et al., 2002). Further, the cytotoxic awajanomycin from *Acremonium* species, cytochalasins from *Rhinocladiella*, and colletotrilactams A–D from endophytic *Colletotrichum gloeosporioides* all exemplify fungal production of higher order lactams (Wagenaar et al., 2000; Jang et al., 2006; Wei et al., 2016). Such lactam production among fungi diversifies xenobiotic composition in soil and may contribute to the discovery of new valuable antibiotics.

### Antifungal Lactams

**FIGURE 3:**
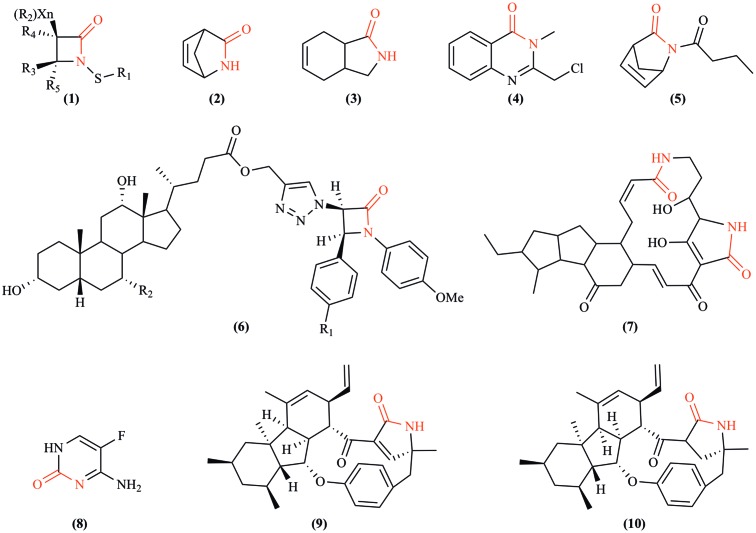
Fungicidal or fungistatic lactams. **(1)** *N*-thiolated β-lactams, artificial compounds that possess antifungal activity against *Candida* and other fungi by exerting powerful cytostatic effects that disrupt the structural integrity of cytoplasmic membranes (O’Driscoll et al., 2008); **(2)** Vince lactam, a versatile artificial chemical intermediate used in organic and medicinal chemistry that shows fungistatic effects against *Botrytis cinerea*, *Penicillium citrium*, and *Aspergillus glaucus* (Walczak et al., 2014); **(3)** (±)-*cis*-3-azabicyclo[4.3.0]non-7-en-2-one, an artificially synthesized compound that is also fungistatic to the same three species as Vince lactam (Walczak et al., 2014); **(4)** 2-chloromethyl-3-methyl-4(3H)-quinazolinone, exhibiting antifungal activity against *Fusarium oxysporum* and *Macrophomina sorgina* (Reddy et al., 2010); **(5)** (±)-2-butyl-2-azabicyclo[2.2.1]hept-5-en-3-one, an artificially synthesized compound that moderately inhibits the growth of *A. glaucus*; **(6)** 1,2,3-triazole-linked β-lactam-bile acid conjugates (R_1_ = H or Cl, R_2_ = H or OH), a group of artificially synthesized compounds that inhibit the growth of *F. oxysporum*, *Candida albicans*, *Cryptococcus neoformans*, *Benjaminiella poitrasii*, *Yarrowia lipolytica* (Vatmurge et al., 2008); **(7)** maltophilin, produced by a ubiquitous free-living bacterium *Stenotrophomonas maltophilia*, which demonstrates inhibitory effects against several Ascomycetes, such as *Aspergillus terreus*, *B. cinerea*, *C. albicans*, *Fusarium solani*, etc. (Jakobi et al., 1996); **(8)** flucytosine, an effective antifungal compound indicated for the treatment of serious infections caused by susceptible strains of *Candida* or *Cryptococcus neoformans* (Cuenca-Estrella et al., 2001); **(9, 10)** pyrrocidine A and B, respectively, broad spectrum antibiotics produced by *Sarocladium zeae* (He et al., 2002).

In addition to the fungal production of bactericidal lactams, emerging evidence indicates that certain atypical lactams can be fungistatic or fungicidal regardless of their origins (**Figure 3**; Brakhage et al., 2009). Novel monocyclic *N*-thiolated β-lactams revealed varying degrees of *in vitro* antifungal activity against seven *Candida* species (O’Driscoll et al., 2008). The fungistatic mode of action against *Candida* was postulated to simulate what was observed against *Staphylococcus aureus*, where these lactams diffused through the cell membrane and interacted covalently with an unknown and possibly evolutionarily conserved target. Two synthetic azetidin-2-one compounds showed moderate antifungal activity against *Botrytis cinerea*, *Colletotrichum lindemuthianum*, and the oomycete *Phytophthora infestans* (Arnoldi et al., 1990). The previously mentioned pyrrocidine A and B from *S. zeae* are antagonistic to kernel rotting fungi including *Aspergillus flavus* and *F. verticillioides* (Wicklow et al., 2005). Interestingly, pyrrocidine A differs from B only in that it possesses a double bond in the γ-lactam ring, and pyrrocidine A shows inhibition at a lower concentration than does B, implying the relevance of the lactam ring to antibiosis. Alternatively, structural conformation changes conveyed by the single vs. double bond could potentially play a role in the observed differential toxicity. Recent studies on synthetic bicyclic lactam analogs of natural plant derived lactones have also revealed their fungistatic effects against *B. cinerea*, *Penicillium citrinum*, and *Aspergillus glaucus* (Walczak et al., 2014). For example, by replacing an oxygen atom with nitrogen in the five-membered ring during a heteroatom analysis of *cis*-3-oxabicyclo-[4.3.0]non-7-en-2-one, a novel γ-lactam compound was created with a significant increase in antifungal activity (Walczak et al., 2014). These discoveries should stimulate further exploration of antifungal lactams and their modes of action.

## β-Lactamases

### Lactam Resistance

**FIGURE 4:**
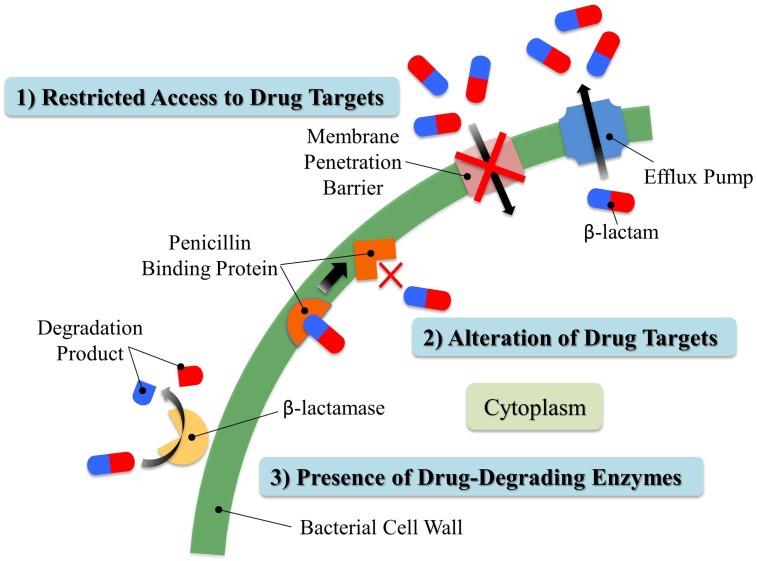
Three major resistant mechanisms present in bacteria against β-lactam antibiotics. Bacteria can develop resistance to β-lactams by (1) restricting their access to penicillin binding proteins; (2) altering penicillin binding proteins to avoid being recognized by β-lactams; (3) producing β-lactamases. Generally speaking, Gram-negative bacteria retain β-lactamases in the periplasmic space between inner and outer membranes. In contrast, Gram-positive bacteria do not possess an outer membrane, and they usually release β-lactamases to the extracellular environment. Fine details of the bacterial membranes and peptidoglycan layer are not shown in this simplified drawing.

The spread of antibiotic resistance among bacteria is one of today’s major world health concerns (Berendonk et al., 2015). In fact, many current publications in the popular press are predicting the end of the age of antibiotics in the near future (Sun and Dennis, 2016), and the World Health Organization recently held a conference on the subject entitled “The end of antibiotics?” Natural sources and clinical/agricultural overuse of antibiotics impose selection pressure for antibiotic resistance, leading to a rise in the number of resistant microbes and the spread of resistant genes regardless of their origins (Allen et al., 2010; Chang et al., 2015). Currently, three major mechanisms have been proposed to generate resistance to β-lactam antibiotics (**Figure 4**): (1) restricted access to drug targets either by (a) preventing drug entry or (b) enhanced drug efflux (Li et al., 1994), (2) alteration of drug targets (Malouin and Bryan, 1986), or (3) the presence of drug-degrading enzymes (Fernandes et al., 2013). Moderate lactam resistance may be developed by intragenic recombination, where genetically distinct alleles occasionally are produced. Such events generate, for example, new alleles of mosaic transpeptidase (penicillin target protein) genes with low penicillin-binding affinities (Zhang et al., 1990; Campos et al., 1992). HGT was proposed decades ago as another means of acquisition of lactam resistance. HGT appears responsible for the spread of both resistance-conferring transpeptidases and plasmid-encoded β-lactamases contributing to high-level lactam resistance and the appearance of “superbugs” with resistance to most or all current antibiotic therapies (Dowson et al., 1990; Coffey et al., 1993; Weldhagen, 2004; Davies and Davies, 2010).

### Bacterial β-Lactamases

β-Lactamase enzymes are the most common mechanism of resistance to β-lactam antibiotics, hydrolyzing the lactam bond in their four-membered ring structures to abolish activity (Livermore, 1998). As these antibiotics are classically active against peptidoglycan cell wall synthesis, the corresponding hydrolytic β-lactamases result in high prevalence of resistant strains and a potential increase in virulence. The first penicillin-hydrolyzing β-lactamase identified was an AmpC cephalosporinase in *Escherichia coli* in 1940, several years before the actual introduction of penicillin into clinical practice (Abraham and Chain, 1940).

Two primary schemes of classifying bacterial β-lactamases have been proposed based on functionality or molecular characteristics (**Table 1**). The functionality classification scheme divides bacterial β-lactamases into three major groups based on inhibitory specificities and the potential requirement of zinc ion for activity (Bush et al., 1995; Frère, 1995). Group 1 includes cephalosporinases that are not well inhibited by active site-directed β-lactamase inhibitors, such as clavulanic acid. Group 2 encompasses β-lactamases that are inhibited by clavulanic acid. Group 3 refers to MBLs that require zinc ions for activity. In addition to conventional hydrolases targeting β-lactams, the MBL superfamily includes lactonases that hydrolyze lactone bonds. A classic example is *N*-acyl homoserine lactonase produced by various bacteria. These lactonases are able to inactivate *N*-acyl homoserine lactones by hydrolyzing the lactone bond, resulting in quenching of bacterial quorum-sensing signaling (Dong et al., 2001; Riaz et al., 2008). The necessity of zinc ions is suspected by the universal presence of a conserved di-nuclear zinc binding site in known lactonases and confirmed by zinc’s essential role during catalytic activity and protein folding (Thomas et al., 2005). The second scheme for classification of β-lactamases utilizes nucleotide and amino acid sequences to divide them into four molecular classes designated A–D (Bush et al., 1995). Enzymes belonging to class A, C, and D act by a serine-based mechanism, often containing Pfam domain PF00144. Those in class B are zinc-based MBLs with Pfam domain PF00753, equivalent to functional Group 3. Serine-based β-lactamases (SBLs) possess conserved motifs S-X-X-K, S/Y-X-N/V, and K-T/S-G in that order, where the serine in the first motif serves as the active site targeting the β-lactam ring. Class B β-lactamases contain a primary zinc-binding motif H-X-H-X-D-H followed by conserved amino acids of Gly, Leu, His, Gly, Asn, and His at specific positions. Except for these conserved amino acids, the rest of their sequences are generally divergent, with greatly differing tertiary structures and catalytic efficiencies (Ehmann et al., 2012).

**Table 1:** Classification of bacterial β-lactamases.

Functional classification	Molecular classification	Inhibition by clavulanic acid	Zinc requirement	Function
Group 1	Class C	No	No	Cephalosporinase
Group 2				
2a	Class A	Yes	No	Penicillinases
2be	Class A	Yes	No	Extended-spectrum β-lactamases
2br	Class A	Yes	No	Inhibitor-resistant TEM-derivative enzymes
2c	Class A	Yes	No	Carbenicillinase
2d	Class A/D	Yes	No	Cloxacilanase
2e	Class A	Yes	No	Cephalosporinase
2f	Class A	Yes	No	Carbapenemase
Group 3	Class B	No	Yes	Metalloenzyme

### Fungal Lactamases

**FIGURE 5:**
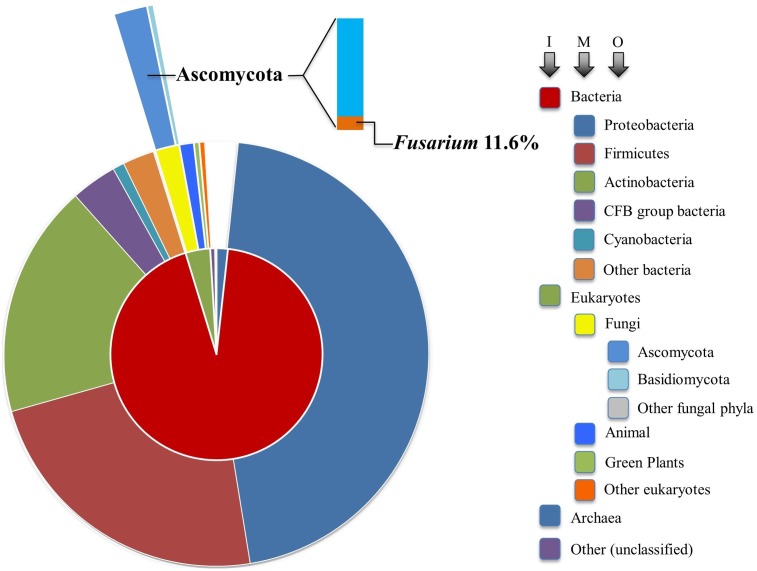
Sunburst visualization of β-lactamase gene distribution by major taxon. Each node of the taxonomic hierarchy is represented as a separate arc, arranged radially with the domains at the center and the phyla arrayed around the outermost ring. The area of each arc is proportional to the number of β-lactamases reported in the NCBI protein database. I, M, and O refer to inner, middle, and outer arcs. The frequency of *Fusarium* lactamases among the Ascomycota is denoted in bar chart form.

Interestingly, genes encoding proteins with β-lactamase homology are widely distributed across major taxa. As of March 1, 2017, there were 1,096,469 manually and computationally annotated β-lactamase encoding genes reported in the National Center for Biotechnology Information (NCBI) protein database across all kingdoms of life. As depicted in **Figure 5**, 93% of them (1,021,177 genes) lie in the domain Bacteria. Although non-bacterial lactamases share similarities with those found in bacteria, less than 1% have been functionally characterized. It is very likely that many non-bacterial “β-lactamases” are not involved in degrading classic β-lactams, so we will refer to them simply as lactamases below. Interestingly, of the roughly one million database entries with suspected lactamase homologs, 14,923 genes were found in fungi, which represents approximately half of the eukaryotic total (29,804 genes). Due to ever-increasing affordability and ease of sequencing, newly identified genes encoding putative lactamases are being added at an accelerating rate to databases.

Even though a large number of fungal genes have been identified that putatively encode lactamases with Pfam domains PF00144 or PF00753 similar to bacteria, only a few gene products have confirmed functions. For example, *Saccharomyces cerevisiae* possesses a small core set of highly conserved enzymes with lactamase domains, but they tend to have specialized functions not involving lactam hydrolysis (**Table 2**). The essential gene *TRZ1* from *S. cerevisiae* encodes tRNase Z, involved in RNA processing (Chen et al., 2005; Zhelkovsky et al., 2006). The essential endonuclease YSH1 in *S. cerevisiae* contains a MBL domain and plays key roles in pre-mRNA 3′ end formation, cooperating with other cleavage factors (Stumpf and Domdey, 1996). Filamentous fungi, including *Fusarium* (**Table 2**), possess *YSH1* and *TRZ1* orthologs in their genomes. Non-essential fungal lactamases appear to have diversified functions, not restricted to nucleases. The non-essential BDS1 in *S. cerevisiae*, presumably horizontally acquired from bacteria, possesses an MBL domain and functions as a sulfuric ester hydrolase (Hall et al., 2005). A discrete MBL type thioesterase in *Aspergillus fumigatus* was found to be required for biosynthesis of endocrocin, a simple anthraquinone commonly identified in fungal extracts (Lim et al., 2012). Asperthecin, a polyketide anthraquinone pigment, is produced by certain *Aspergillus* species (Howard and Raistrick, 1955), and disruption of the asperthecin biosynthetic gene cluster in *Aspergillus nidulans* revealed that a lactamase assisted the adjacent polyketide synthase to hydrolyze an aromatic polyketide into endocrocin-9-anthrone (Szewczyk et al., 2008). LovD, a SBL containing the PF00144 motif, in *Aspergillus terreus* was essential for lovastatin biosynthesis, and it was also later described to be involved in synthesizing simvastatin, a lipid-lowering agent, by acting on a protein-bound acyl substrate (Kennedy, 1999; Jiménez-Osés et al., 2014). Through proteomic studies on both weakly and highly aggressive *Verticillium dahliae* isolates, it was inferred that a β-lactamase family protein might act as a pathogenicity factor that is recognized by the host plant immune system as an elicitor (El-Bebany et al., 2010). Thus, the functional diversity of fungal lactamases is evident despite limited studies.

**Table 2:** *Saccharomyces cerevisiae* lactamase orthologs in three *Fusarium* species.

*Saccharomyces cerevisiae* lactamase genes^∗^			*Fusarium* orthologs		
Systematic name	Standard name	Function	Fv FVEG_	Fo FOXG_	Fg FGSG_
YDR272W	*GLO2*	Hydroxyacylglutathione hydrolase GLO2	08018/ 16907	01652/ 12249	13072
YKR079C	*TRZ1*	tRNase Z	05485	02309	06635
YLR277C	*YSH1*	Cleavage polyadenylation factor subunit YSH1	14723	17946	00819
YMR137C	*PSO2*	Pso2p nuclease	00815	00696	00361
YOL164W	*BDS1*	Sulfuric ester hydrolase	N/I^∗∗^	N/I	N/I
YOR040W	*GLO4*	Hydroxyacylglutathione hydrolase GLO4	08018/ 16907	01652/ 12249	13072
YPL103C	*FMP30*	*N*-acetylphosphatidylethanolamine-hydrolyzing phospholipase D	03849	05981	09261

*^∗^Saccharomyces cerevisiae has seven lactamase genes, two of which (*TRZ1* and *YSH1*) are essential. ^∗∗^N/I represents no orthologs identified in these three *Fusarium* species. Fv, *F. verticillioides*; Fo, *F. oxysporum*; Fg, *F. graminearum**.

Recent literature has shown that two related fungal lactamases function in xenobiotic hydrolysis, similar to bacterial counterparts. In fact, our interest in fungal lactamases stems from the observation that the gene FVEG_08291 in *F. verticillioides* encodes a lactamase designated MBL1 that is responsible for the degradation of 2-benzoxazolinone (BOA) (Glenn et al., 2016). A similar but non-orthologous MBL in *F. pseudograminearum* (FPSE_08124) was also shown to be responsible for BOA degradation (Kettle et al., 2015a). BOA is a γ-lactam phytochemical produced by select graminaceous crops that is implicated in resistance to insect herbivory and microbial pathogens. The enzymatic capacity of *Fusarium* species to hydrolyze BOA is suggested to enhance colonization of the host, thus increasing the frequency and abundance of the species (Saunders and Kohn, 2008; Saunders et al., 2010). Interestingly, *MBL1* is part of a gene cluster that is up-regulated in response to BOA, and this cluster, called the *FDB1* cluster, was also observed in the other maize pathogens *Fusarium subglutinans* and *Colletotrichum graminicola* (Glenn et al., 2016). The highly conserved synteny of the *FDB1* cluster between these fungi suggests *C. graminicola* acquired the cluster from *Fusarium* by HGT, and that the maize host and its phytochemicals, notably BOA and related lactams, are driving factors influencing the evolution and genomic content of these fungi.

## *Fusarium* Lactamases

### *Fusarium* Lactamase Analysis as a Paradigm?

**FIGURE 6:**
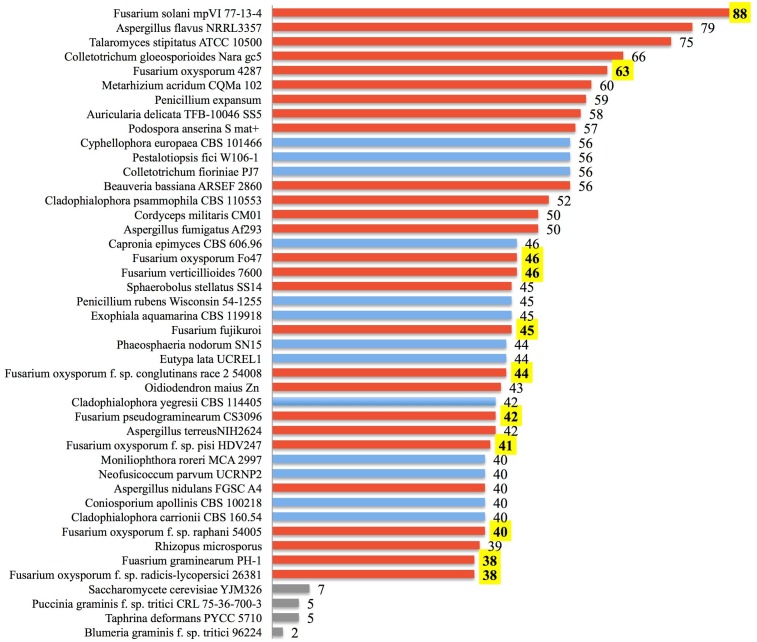
Lactamase genes are abundant within some fungi. The number of annotated ORFs possessing a lactamase domain is shown for the top 40 fungi among all annotated fungal species identified from the NCBI Protein Database. Species are ranked by the abundance of lactamases. Fungi known to be soil-borne are displayed with red bars, those not clearly cited in literature as soil-borne fungi are in blue, and yeast along with selected obligate plant pathogens are in gray. Numbers of *Fusarium* lactamases are highlighted in yellow.

Our analysis suggests that soil-borne fungi tend to possess more lactamase encoding genes compared with the minimal sets from fungi predicted to live in environments of relatively low microbial diversity (**Figure 6**). Broadly distributed in soil, *Fusarium* species are likely in competition with diverse microbes and are presumably often exposed to xenobiotic compounds. Frequent confrontation with competing microorganisms inhabiting overlapping ecological niches is expected to hone genetic determinants of xenobiotic resistance. *Fusarium* interactions with soil competitors are complex, involving nutrient competition and chemical warfare (Kinkel et al., 2012). As noted in **Figure 5**, the majority (84.3%) of sequenced fungal lactamase encoding genes are from the phylum Ascomycota (12,771 genes), 11.6% of which belonged to the genus *Fusarium* (1479 genes). There were, on average, 37 lactamase encoding genes per *Fusarium* species, as opposed to 15 per species among non-*Fusarium* fungal genomes. The species noted with the highest number of lactamase encoding genes, 88, was the soil limited root pathogen *Fusarium solani* (**Figure 6**). Thus, we further propose that the abundance of *Fusarium* lactamases is likely integral to the success of this genus as a soil competitor. Further analysis of the global and individual roles of lactamases is important for more fully understanding *Fusarium* biology and its ecological interactions.

### Detailed Analysis of *F. verticillioides* Lactamases

To better understand molecular characteristics of *Fusarium* lactamases, we cataloged the complete set of lactamase encoding genes from three representative and pathogenically important sequenced *Fusarium* genomes, *F. verticillioides* 7600 (*Fv*), *Fusarium oxysporum* 4287 (*Fo*), and *Fusarium graminearum* PH-1 (*Fg*), via homology-based protein reciprocal BLAST and bacterial β-lactamase HMMER sequence logo scanning (Wheeler and Eddy, 2013). We identified 46 lactamase domain-containing genes in *Fv*, 63 in *Fo*, and 38 in *Fg*, as listed in **Table 3** by predicted enzymatic mechanisms (MBLs and SBLs). PSI-BLAST of each lactamase encoding gene in *F. verticillioides* helped uncover distant homologs and confirm domain integrity. Interestingly, some predicted SBL gene annotations (FVEG_03300, FVEG_14143, FVEG_15166, FVEG_17257, FVEG_17258) were missing core catalytic serine motifs or possessed only part of the conventional β-lactamase folds. Thus, these five genes were further evaluated for their open reading frames using the FGENESH program from Softberry (http://www.softberry.com) to refine gene predictions. Reannotated sequences suggested FVEG_17257 and FVEG_17258 should be merged as one lactamase encoding gene, while FVEG_03300, FVEG_14143, and FVEG_15166 remained unchanged, still missing the core serine and lacking canonical amino acids at the majority of conserved sites. These three were thus excluded from later syntenic and phylogenetic analyses. PSI-BLAST of MBLs in *F. verticillioides* also predicted several members could be involved in metabolizing RNA (FVEG_05485, FVEG_11466, FVEG_14723), degrading lipids (FVEG_11923, FVEG_03849), repairing DNA (FVEG_00815, FVEG_04252), and hydrolyzing hydroxylacyl glutathione (FVEG_08018, FVEG_16907), which also require zinc ions for appropriate functions. See also **Table 2**.

**Table 3:** β-lactamase domain-containing genes in three *Fusarium* genomes.

Species	Accession number					
	MBL (FVEG_)					SBL (FVEG_)				
	00815	03849	04252	05261	05485	01581	01641	01651	03303	05963
	05734	05854	08018	08291	09433	09854	09904	12457	12760	13172
*F. verticillioides* 7600	11466	11838	11923	12159	12288	05685	04555	03457	10996	01795
	12347	12526	12637	13253	13366	03300	14143	10753	10740	09057
	13675	14723	14874	16907		15166	17257			

	**MBL (FOXG_)**					**SBL (FOXG_)**				
	00696	01652	02309	02559	03706	02097	02670	02810	02811	02821
	03847	03877	04928	06402	06970	03275	03924	**05576**	05981	**07628**
	07119	08819	08964	12116	12249	08711	10409	10814	10816	10887
***F. oxysporum* f. sp. *lycopersici* 4287**	12727	12984	13156	13240	13402	10911	10955	**12166**	12179	**13106**
	**14524**	15197	15260	15319	15773	13918	**14363**	15115	**15119**	15429
	15776	16562	17598	17946	18400	17393	**18438**	18914	21695	22119
	20403					22149	22249			

	**MBL (FGSG_)**					**SBL (FGSG_)**				
	00079	00361	00819	03085	04727	00024	02452	02875	**03050**	03364
	05331	06635	07959	10497	10653	**04656**	04809	04813	05706	07314
***F. graminearum* PH-1**	10795	11082	11291	11553	13072	07538	07702	**07996**	08136	08476
	13173					09143	09261	**10287**	10497	11664
						13212	13439			

*MBL, metallo- β-lactamases; SBL, serine-based β-lactamases. Bolded accession numbers are those with signal peptides identified by SignalP 4.1 Server, suggesting likely secretion (Petersen et al., 2011)*.

**FIGURE 7:**
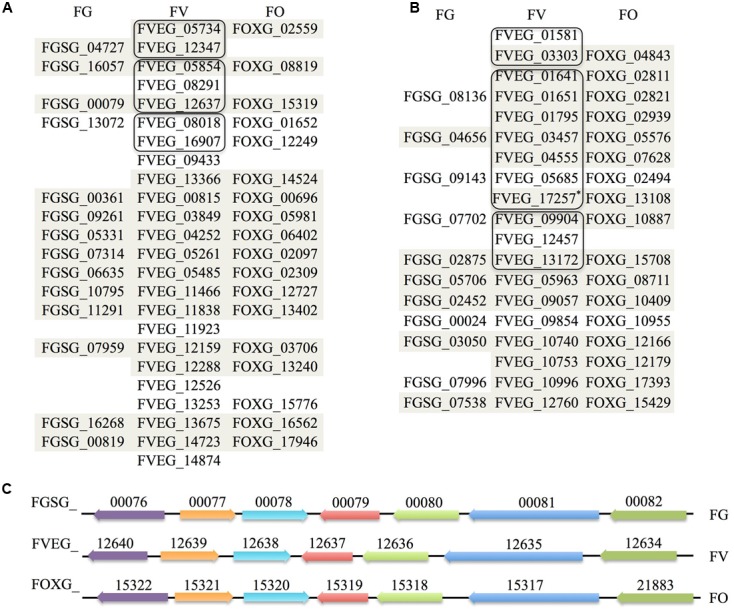
Orthological and syntenic analysis of *Fusarium verticillioides*, *Fusarium graminearum*, and *F. oxysporum* lactamase genes. Each column lists predicted β-lactamase orthologs in the three *Fusarium* species. Those sharing synteny of the adjacent 20 kb regions are shaded (10 kb upstream and 10 kb downstream). Amino acid sequences of *F. verticillioides* β-lactamases sharing more than 40% sequence identity are considered as paralogs and grouped in outlined boxes. **(A)** MBLs synteny. **(B)** SBLs synteny. The modified nucleotide sequence merging FVEG_17257 and FVEG_17258 based on FGENESH prediction was renamed FVEG_17257^∗^ here and used for syntenic studies. **(C)** Demonstration of synteny of genes flanking β-lactamases exemplified by FVEG_12637, which represents part of the *FDB2* gene cluster essential for the biotransformation of 2-benzoxazolinone (Glenn and Bacon, 2009; Glenn et al., 2016). Orthologs are shown in the same color with accession numbers above, and direction of arrows represents the orientation of genes.

**FIGURE 8:**
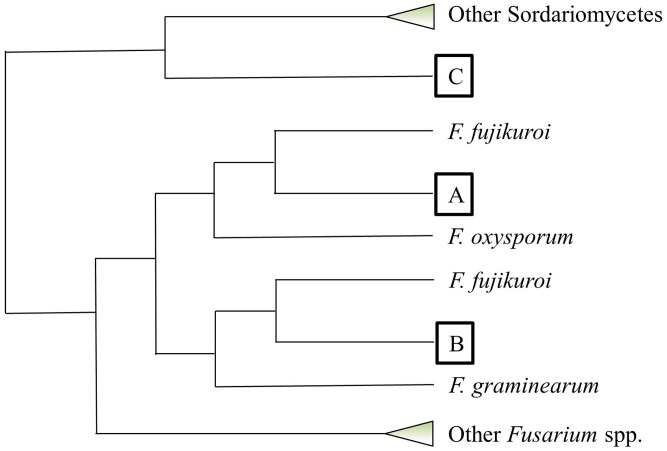
Phylogenetic placement of predicted *Fusarium verticillioides* lactamase genes based on amino acid sequence alignment. This cartoon summarizes the phylogenetic pattern of lactamase genes with intact core motifs in *F. verticillioides*. Each query lactamase amino acid sequence was searched for its top 50 homologs using BLASTP in NCBI. Neighbor-joining trees using the Jukes–Cantor genetic distance model were constructed by Geneious Tree Builder (Ver. 8.1) for each individual homology search. All tree topographies complied with the configuration such that each *F. verticillioides* lactamase fell into one of three positions marked as A, B, and C. Collapsed clades are shown as triangles.

*Fv*-oriented syntenic studies were performed such that corresponding orthologs and adjacent genes in *Fg* and *Fo* were examined. In terms of species phylogeny, *Fv* is more closely related to *Fo* than *Fg*. Thus, we naturally expected more orthologs identified in *Fo*. Except for those *Fv* lactamase encoding genes with no orthologs in the other two species, the rest of the genes generally retained syntenic clusters in the *Fg* and/or *Fo* genomes (**Figure 7**). Phylogenetic evaluation of 41 *Fv* genes having the core lactamase motifs revealed a high consistency with species evolution (**Figure 8**), where 37 genes fall into position A, clustering with orthologs in *Fusarium fujikuroi* in the respective phylograms. This suggests that *Fv* lactamases are most similar to those annotated in closely related species compared with other relatively distant species. Lactamase encoding genes in *Fusarium* species generally form a clade distinct from other Sordariomycetes. Interestingly, only 29% of the *Fv* MBLs had evidence of paralogy (**Figure 7**), whereas 63% of the *Fv* SBLs appeared to have paralogs. This suggests the two types of lactamases may have different evolutionary pressures impacting duplication and diversification. Only six *Fv* lactamase encoding genes lack possible orthologs in both *Fo* and *Fg* (**Figure 7**). Collectively the data indicate that some lactamase encoding genes originated before the divergence of *Fusarium* species, resulting in greater sequence diversity accompanying species divergence. Interestingly, FVEG_12347 was the only gene in the phylogenetic position B (**Figure 8**), suggesting that it is similar to *Fg* ortholog FGSG_04727 and lacks an ortholog in *Fo* (**Figure 7**). FVEG_08291, FVEG_09433, and FVEG_12457 notably exhibited more similarities to orthologs in other Sordariomycetes rather than in closely related *Fusarium* species. These fall into phylogenetic position C and are thus good candidates for HGT derivation. The FVEG_08291 protein sequence possessed 85% identity to an ortholog in *C. graminicola* (NCBI Reference Sequence: XP_008099767.1), another Sordariomycetes pathogen of maize, surpassing homology to other related genes in *Fusarium* species. This is the *MBL1* gene noted above as part of the *FDB1* cluster conferring resistance to BOA. A similar case was observed for FVEG_09433, where it was more closely related to orthologs in *C. graminicola* and other genera than to those of most other *Fusarium* species, even though there are apparent orthologs in *Fusarium mangiferae* (GenBank ID: CVL02248.1) and *F. fujikuroi* (GenBank ID: CCT69225.1). One possible explanation is that FVEG_09433 was introduced to *Fusarium* species within the *F. fujikuroi* species complex prior to the divergence of these three species, but its orthologs among other species of the complex were somehow lost. The serine-based lactamase encoded by FVEG_12457 was most similar to its orthologs in *A. terreus* (NCBI Reference ID: XP_001217058.1) and *Penicillium roqueforti* (GenBank ID: CDM29397.1) with a sequence identity of over 70%, exceeding the 60% average identity among related *Fusarium* genes.

**FIGURE 9:**
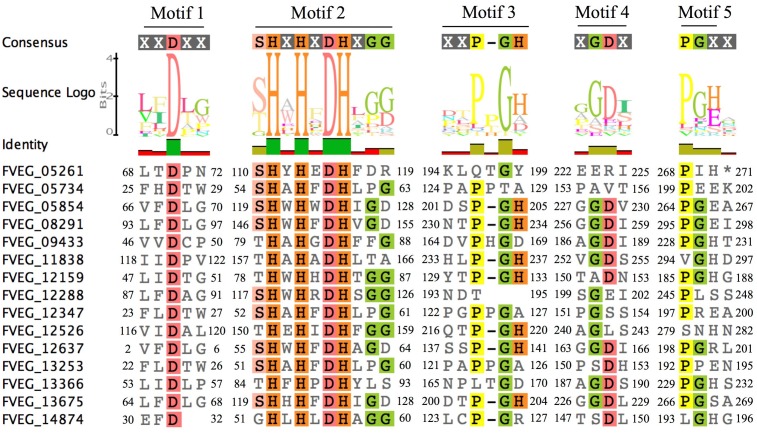
Sequence alignment of motifs within *Fusarium verticillioides* metallo-β-lactamase proteins that are presumably associated with lactam hydrolysis. Amino acids matching at least 50% of all sequences are highlighted.

**FIGURE 10:**
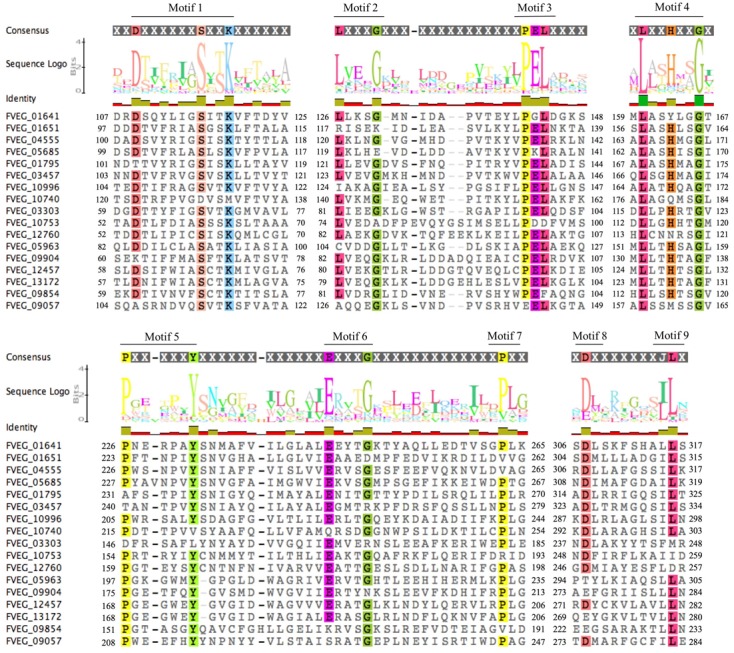
Sequence alignment of motifs within *Fusarium verticillioides* serine-based β-lactamases proteins that are presumably associated with lactam hydrolysis. Amino acids matching at least 75% of all sequences included were highlighted.

Multiple Alignment using Fast Fourier Transform (MAFFT) analysis of presumed hydrolysis-related lactamase protein sequences was performed separately for MBLs and SBLs, presenting two distinctive patterns of conserved motifs (**Figures 9**, **10**). Although these *Fv* lactamases exhibited considerable sequence diversity, conserved motifs were still observed. As to MBLs, the conserved motif His-X-His-X-Asp-His-X-Gly resembled that in classic bacterial MBLs (**Figure 9**). However, compared to typical cases in bacteria, the overall conserved motif pattern is different in *Fv* MBLs, and additional motifs were identified, including Pro-X-Gly-His in Motif 3, Gly-Asp in Motif 4, and Pro-Gly in Motif 5 (**Figure 9**). A retrospective scrutiny of conserved motifs of bacterial PSI-BLAST hits revealed that these sites in *Fv* lactamases are also present in certain bacterial β-lactamases (data not shown). *Fv* SBLs demonstrate an interesting molecular pattern that is not present in bacteria (**Figure 10**). A total of nine motifs were identified in all intact *Fv* SBLs that are predicted to be hydrolysis-associated. Besides the catalytic core motif shared with bacteria (Ser-X-X-Lys as Motif 1), *Fv* lactamases contain conserved amino acids Leu-X-X-X-Gly in Motif 2, Pro-Glu-Leu in Motif 3, Leu-X-X-His-X-X-Gly in Motif 4, Pro-X-X-X-X-X-X-Tyr in Motif 5, Glu-X-X-X-Gly in Motif 6, a single conserved amino acid Pro in Motif 7, the Asp in Motif 8, and the Leu in Motif 9. However, none of these motifs (Motifs 2–9) are represented in bacterial species.

**FIGURE 11:**
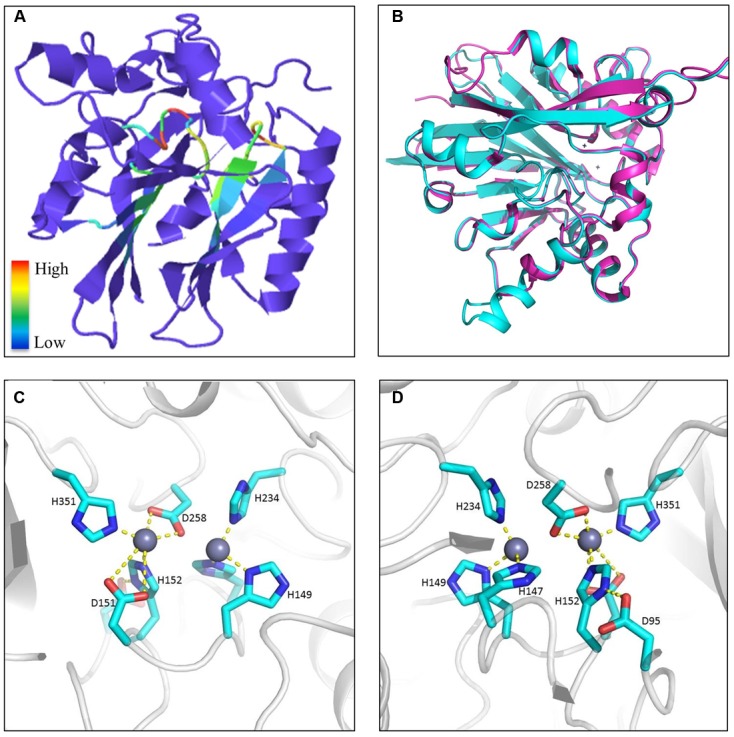
Example of metallo-β-lactamase tertiary structures. **(A)** Predicted protein structure of FVEG_08291 exemplifying a general tertiary structure of metallo-β-lactamases. Motifs are color-coded according to relative conservation as shown in the scale bar. **(B)** Protein superimposition of FVEG_08291 and the 2R2D chain C from the Protein Data Bank (http://www.ebi.ac.uk/pdbe/), where cyan represents FVEG_08291 and magenta represents the 2R2D chain C. Front **(C)** and rear **(D)** view of proposed interactions between zinc ions and conserved MBL amino acids in FVEG_08291.

Phyre2 predictions of tertiary structures reflected an interesting discovery that the majority of *Fv* MBLs were similar to bacterial β-lactamases, presenting a α–β/β–α sandwich structure composed of two β sheets at the core and α helices on the external surfaces (Kelley et al., 2015). Those conserved residues are generally located at flexible loops connecting different secondary structures. It can be inferred that the spatial adjacency of histidines would facilitate the coordination of zinc ions and that the aspartic acid residues participate in the hydrolysis reaction. As exemplified in **Figure 11**, FVEG_08291 was predicted to have the signature sandwich conformation with a flap structure (the flexible mobile loop), which is situated at the bottom of a wide shallow groove between two β-sheets (**Figure 11A**). This structure has proven to be critical in substrate binding in bacteria (Materon and Palzkill, 2001). Superimposition of protein structures revealed that FVEG_08291 resembles a quorum-quenching lactonase (AiiB) from *Agrobacterium tumefaciens* (**Figure 11B**). The conserved zinc-coordinated residues on the flexible loop as well as the easily accessible groove placement suggest the potential to accommodate various lactam or lactone molecules (**Figures 11C,D**). Other conserved amino acids not directly predicted to be associated with catalytic reactions may be involved in structure maintenance or substrate recognition, and overall the catalytic mechanisms of fungal lactamases require further exploration.

## Conclusion and Future Directions

The complexity of soil environments, particularly those with nutrient-driven competition in the rhizosphere, has led to diverse organisms capable of antimicrobial activity. Plants also contribute to rhizospheric antimicrobial content, either proactively (phytoanticipins), or reactive to pathogen contact (phytoalexins) (Morrissey and Osbourn, 1999; Kato-Noguchi et al., 2008). Thus, the soil environment contains high antibiotic diversity including β-lactams, tetracyclines, sulfonamides, aminoglycosides, imidazoles, etc. (Thiele-Bruhn, 2003). Competitive relationships among soil microflora exert selective pressure on genes for antibiotic production and resistance. These genes in turn shape microbial populations and diversity, largely through development of antibiotic resistance mechanisms, such as the enzymatic degradation of β-lactam-containing compounds. Xenobiotic degradation in soil is propelled by enzymatic processes such as hydrolysis, oxidative decarboxylation, and hydroxylation (Chen et al., 1997; Mcgrath et al., 1998; Al-Ahmad et al., 1999; Halling-Sørensen, 2000; Thiele-Bruhn, 2003). Interestingly, functional metagenomics have revealed that, as the major resistance source against β-lactams, β-lactamase encoding genes were abundant even in undisturbed soil absent of anthropogenic selective pressure, contributing to a massive reservoir for genetic exchange among soil microflora (Allen et al., 2009).

**FIGURE 12:**
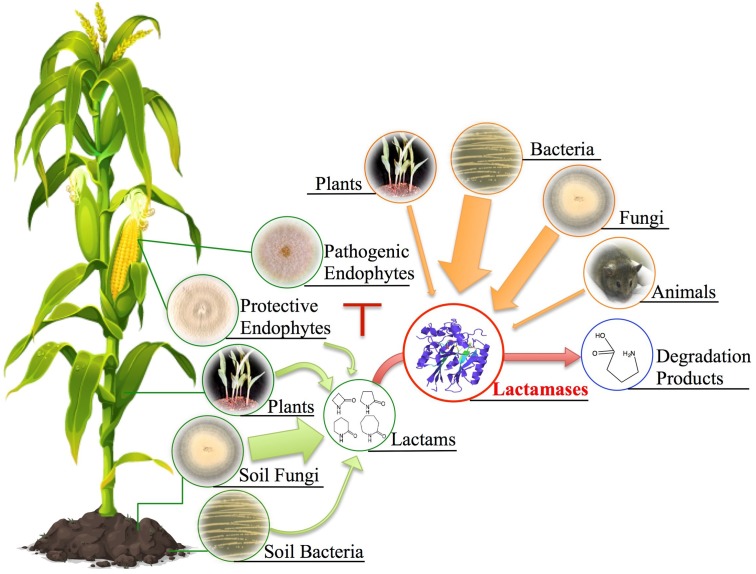
Potential ecological sources of lactam compounds and lactamases. Soil fungi, bacteria, plants, and protective endophytes (e.g., *Sarocladium zeae*) have all been documented to produce lactam-containing compounds. The antibiotic characteristic of these lactams has been implied to be associated with ecological niche competition. Plants, bacteria, fungi, and even animals have developed corresponding hydrolytic lactamases to combat antibiosis. To expand the conventional examples of bacterial β-lactamases, we propose a more generic ecological model linking lactam production with possible hydrolytic functions of organismal lactamases given their universal presence across different kingdoms. Note that arrow width depicts predicted relative abundance based on genomic frequencies.

Given our examination of fungal hydrolytic lactamases, we propose an ecological model (**Figure 12**) centering on the production and function of both lactams and lactamases produced by plants, bacteria, and fungi. We expand the conventional focus beyond that of solely bacterial β-lactamases and instead propose a more generic ecological model linking lactam production with hydrolytic functions of organismal lactamases. Lactam antibiotics presumably benefit their producers by securing ecological niches, whereas numerous lactam producers have also developed hydrolytic lactamases postulated to combat antibiosis. For example, soil-associated fungi typically possess more lactamase encoding genes than those from environments with lower microbial diversity since soil environments contain significant antibiotic diversity. Analysis of *Fusarium* species provides the foundation for our hypothesis that soil fungi frequently utilize lactamases in detoxification of xenobiotics, especially given *Fusarium* species’ wide soil distribution, lactamase-rich genomes, and recent functional characterization of lactamase encoding genes. The general abundance and persistence of lactamase genes in fungal genomes suggests a significant role for these enzymes in the soil environment, presumably in protection from many as yet unknown xenobiotics. We have generated a large set of lactamase mutants in *F. verticillioides* and are conducting transcriptional and phenotypic analyses upon exposure to various lactam compounds in order to more thoroughly evaluate the role and activity of these lactamases, thus broadening our appreciation of both the lactam compounds and corresponding lactamases in terms of their diversity and impact on both bacterial and fungal communities. This work also has the potential to broaden our appreciation of environmental sources of antimicrobial resistance to include both bacteria and fungi, especially with regard to use of antibiotics in agriculture.

## Author Contributions

All authors listed have made a substantial, direct and intellectual contribution to the work, and approved it for publication.

## Conflict of Interest Statement

The authors declare that the research was conducted in the absence of any commercial or financial relationships that could be construed as a potential conflict of interest.

## References

[B1] Abraham E. P. Chain E. (1940). An enzyme from bacteria able to destroy penicillin. 1940. *Rev. Infect. Dis.* 10 677–678. 10.1038/146837a0 3055168

[B2] Agrawal A. A. (2011). Current trends in the evolutionary ecology of plant defence. *Funct. Ecol.* 25 420–432. 10.1111/j.1365-2435.2010.01796.x

[B3] Al-Ahmad A. Daschner F. D. Kümmerer K. (1999). Biodegradability of cefotiam, ciprofloxacin, meropenem, penicillin G, and sulfamethoxazole and inhibition of waste water bacteria. *Arch. Environ. Contam. Toxicol.* 37 158–163. 10.1007/s002449900501 10398765

[B4] Allen H. K. Donato J. Wang H. H. Cloud-Hansen K. A. Davies J. Handelsman J. (2010). Call of the wild: antibiotic resistance genes in natural environments. *Nat. Rev. Microbiol.* 8 251–259. 10.1038/nrmicro2312 20190823

[B5] Allen H. K. Moe L. A. Rodbumrer J. Gaarder A. Handelsman J. (2009). Functional metagenomics reveals diverse beta-lactamases in a remote Alaskan soil. *ISME J.* 3 243–251. 10.1038/ismej.2008.86 18843302

[B6] Arnoldi A. Cabrini M. R. Farina G. Merlini L. (1990). Activity of a series of beta-lactams against some phytopathogenic fungi. *J. Agric. Food Chem.* 38 2197–2199. 10.1021/jf00102a019

[B7] Baker E. A. Smith I. M. (1977). Antifungal compounds in winter wheat resistant and susceptible to *Septoria nodorum*. *Ann. Appl. Biol.* 87 67–73. 10.1111/j.1744-7348.1977.tb00660.x

[B8] Berendonk T. U. Manaia C. M. Merlin C. Fatta-Kassinos D. Cytryn E. Walsh F. (2015). Tackling antibiotic resistance: the environmental framework. *Nat. Rev. Microbiol.* 13 310–317. 10.1038/nrmicro3439 25817583

[B9] Bozdogan B. Appelbaum P. C. (2004). Oxazolidinones: activity, mode of action, and mechanism of resistance. *Int. J. Antimicrob. Agents* 23 113–119. 10.1016/j.ijantimicag.2003.11.003 15013035

[B10] Brakhage A. A. Schroeckh V. (2011). Fungal secondary metabolites - strategies to activate silent gene clusters. *Fungal Genet. Biol.* 48 15–22. 10.1016/j.fgb.2010.04.004 20433937

[B11] Brakhage A. A. Thön M. Spröte P. Scharf D. H. Al-Abdallah Q. Wolke S. M. (2009). Aspects on evolution of fungal β-lactam biosynthesis gene clusters and recruitment of trans-acting factors. *Phytochemistry* 70 1801–1811. 10.1016/j.phytochem.2009.09.011 19863978

[B12] Bush K. Jacoby G. A. Medeiros A. A. (1995). A functional classification scheme for β-lactamases and its correlation with molecular structure. *Antimicrob. Agents Chemother.* 39 1211–1233. 10.1128/AAC.39.6.1211 7574506 PMC162717

[B13] Campos J. Carmen Fusté M. Trujillo G. Sáez-Nieto J. Vázquez J. Lorén J. G. (1992). Genetic diversity of penicillin-resistant *Neisseria meningitidis*. *J. Infect. Dis.* 166 173–177. 10.1093/infdis/166.1.173 1351510

[B14] Chang Q. Wang W. Regev-Yochay G. Lipsitch M. Hanage W. P. (2015). Antibiotics in agriculture and the risk to human health: how worried should we be? *Evol. Appl.* 8 240–247. 10.1111/eva.12185 25861382 PMC4380918

[B15] Chen Y. Beck A. Davenport C. Chen Y. Shattuck D. Tavtigian S. V. (2005). Characterization of TRZ1, a yeast homolog of the human candidate prostate cancer susceptibility gene ELAC2 encoding tRNase Z. *BMC Mol. Biol.* 6:12 10.1186/1471-2199-6-12 PMC1156898 15892892

[B16] Chen Y. Rosazza J. P. N. Reese C. P. Chang H. Y. Nowakowski M. A. Kiplinger J. P. (1997). Microbial models of soil metabolism: biotransformations of danofloxacin. *J. Ind. Microbiol. Biotechnol.* 19 378–384. 10.1038/sj.jim.2900409 9451835

[B17] Coffey T. J. Dowson C. G. Daniels M. Spratt B. G. (1993). Horizontal spread of an altered penicillin-binding protein 2B gene between *Streptococcus pneumoniae* and *Streptococcus oralis*. *FEMS Microbiol. Lett.* 110 335–339. 10.1016/0378-1097(93)90125-L 8354467

[B18] Couture R. M. Routley D. G. Dunn G. M. (1971). Role of cyclic hydroxamic acids in monogenic resistance of maize to *Helminthosporium turcicum*. *Physiol. Plant Pathol.* 1 515–521. 10.1016/0048-4059(71)90013-0

[B19] Cuenca-Estrella M. Díaz-Guerra T. M. Mellado E. Rodríguez-Tudela J. L. (2001). Flucytosine primary resistance in *Candida* species and *Cryptococcus neoformans*. *Eur. J. Clin. Microbiol. Infect. Dis.* 20 276–279. 10.1007/PL00011265 11399020

[B20] Davelos A. L. Kinkel L. L. Samac D. A. (2004). Spatial variation in frequency and intensity of antibiotic interactions among streptomycetes from prairie soil. *Appl. Environ. Microbiol.* 70 1051–1058. 10.1128/AEM.70.2.1051-1058.2004 14766588 PMC348876

[B21] Davies J. (2006). Are antibiotics naturally antibiotics? *J. Ind. Microbiol. Biotechnol.* 33 496–499. 10.1007/s10295-006-0112-5 16552582

[B22] Davies J. Davies D. (2010). Origins and evolution of antibiotic resistance. *Microbiol. Mol. Biol. Rev.* 74 417–433. 10.1128/MMBR.00016 20805405 PMC2937522

[B23] Delserone L. M. Matthews D. E. VanEtten H. D. (1992). Differential toxicity of enantiomers of maackiain and pisatin to phytopathogenic fungi. *Phytochemistry* 31 3813–3819. 10.1016/S0031-9422(00)97534-4

[B24] Demain A. L. Elander R. P. (1999). The β-lactam antibiotics: past, present, and future. *Antonie Van Leeuwenhoek* 75 5–19. 10.1023/A:1001738823146 10422578

[B25] Dong Y. H. Wang L. H. Xu J. L. Zhang H. B. Zhang X. F. Zhang L. H. (2001). Quenching quorum-sensing-dependent bacterial infection by an N-acyl homoserine lactonase. *Nature* 411 813–817. 10.1038/35081101 11459062

[B26] Dowson C. G. Hutchison A. Woodford N. Johnson A. P. George R. C. Spratt B. G. (1990). Penicillin-resistant *Viridans streptococci* have obtained altered penicillin-binding protein genes from penicillin-resistant strains of *Streptococcus pneumoniae*. *Proc. Natl. Acad. Sci. U.S.A.* 87 5858–5862. 10.1073/pnas.87.15.5858 2377622 PMC54428

[B27] Duczek L. J. Higgins V. J. (1976). Effect of treatment with the phytoalexins medicarpin and maackiain on fungal growth *in vitro* and *in vivo*. *Can. J. Bot.* 54 2620–2629. 10.1139/b76-282

[B28] Ehmann D. E. Jahić H. Ross P. L. Gu R.-F. Hu J. Kern G. (2012). Avibactam is a covalent, reversible, non–β-lactam β-lactamase inhibitor. *Proc. Natl. Acad. Sci. U.S.A.* 109 11663–11668. 10.1073/pnas.1205073109 22753474 PMC3406822

[B29] El-Bebany A. F. Rampitsch C. Daayf F. (2010). Proteomic analysis of the phytopathogenic soilborne fungus *Verticillium dahliae* reveals differential protein expression in isolates that differ in aggressiveness. *Proteomics* 10 289–303. 10.1002/pmic.200900426 20017145

[B30] Fernandes R. Amador P. Prudêncio C. (2013). β-Lactams: chemical structure, mode of action and mechanisms of resistance. *Rev. Med. Microbiol.* 24 7–17. 10.1097/MRM.0b013e3283587727

[B31] Fleming A. (1929). On the antibacterial action of cultures of a *Penicillium*, with special reference to their use in the isolation of *B. influenzae*. *Br. J. Exp. Pathol.* 10 226–236. 10.1038/146837a0

[B32] Forseth R. R. Fox E. M. Chung D. Howlett B. J. Keller N. P. Schroeder F. C. (2011). Identification of cryptic products of the gliotoxin gene cluster using NMR-based comparative metabolomics and a model for gliotoxin biosynthesis. *J. Am. Chem. Soc.* 133 9678–9681. 10.1021/ja2029987 21612254 PMC3151163

[B33] Frère J. M. (1995). Beta-lactamases and bacterial resistance to antibiotics. *Mol. Microbiol.* 16 385–395. 10.1111/j.1365-2958.1995.tb02404.x 7565100

[B34] Gans J. (2006). Response to comment by Bunge et al. on “Computational improvements reveal great bacterial diversity and high metal toxicity in soil”. *Science* 313 918 10.1126/science.1126853 16917045

[B35] Gardiner D. M. Cozijnsen A. J. Wilson L. M. Pedras M. S. C. Howlett B. J. (2004). The sirodesmin biosynthetic gene cluster of the plant pathogenic fungus *Leptosphaeria maculans*. *Mol. Microbiol.* 53 1307–1318. 10.1111/j.1365-2958.2004.04215.x 15387811

[B36] Glenn A. E. Bacon C. W. (2009). FDB2 encodes a member of the arylamine N-acetyltransferase family and is necessary for biotransformation of benzoxazolinones by *Fusarium verticillioides*. *J. Appl. Microbiol.* 107 657–671. 10.1111/j.1365-2672.2009.04246.x 19302487

[B37] Glenn A. E. Davis C. B. Gao M. Gold S. E. Mitchell T. R. Proctor R. H. (2016). Two horizontally transferred xenobiotic resistance gene clusters associated with detoxification of benzoxazolinones by *Fusarium* species. *PLOS ONE* 11:e0147486 10.1371/journal.pone.0147486 PMC4726666 26808652

[B38] Goh E.-B. Yim G. Tsui W. McClure J. Surette M. G. Davies J. (2002). Transcriptional modulation of bacterial gene expression by subinhibitory concentrations of antibiotics. *Proc. Natl. Acad. Sci. U.S.A.* 99 17025–17030. 10.1073/pnas.252607699 12482953 PMC139263

[B39] González-Lamothe R. Mitchell G. Gattuso M. Diarra M. S. Malouin F. Bouarab K. (2009). Plant antimicrobial agents and their effects on plant and human pathogens. *Int. J. Mol. Sci.* 10 3400–3419. 10.3390/ijms10083400 20111686 PMC2812829

[B40] Grotewold E. (2005). Plant metabolic diversity: a regulatory perspective. *Trends Plant Sci.* 10 57–62. 10.1016/j.tplants.2004.12.009 15708342

[B41] Hall C. Brachat S. Dietrich F. S. (2005). Contribution of horizontal gene transfer to the evolution of *Saccharomyces cerevisiae*. *Eukaryot. Cell* 4 1102–1115. 10.1128/EC.4.6.1102-1115.2005 15947202 PMC1151995

[B42] Halling-Sørensen B. (2000). Algal toxicity of antibacterial agents used in intensive farming. *Chemosphere* 40 731–739. 10.1016/S0045-6535(99)00445-2 10705551

[B43] Harrison C. J. Bratcher D. (2008). Cephalosporins: a review. *Pediatr. Rev.* 29 264–273. 10.1542/pir.29-8-264 18676578

[B44] Hawksworth D. L. (1991). The fungal dimension of biodiversity: magnitude, significance, and conservation. *Mycol. Res.* 95 641–655.

[B45] Hazuda D. Blau C. U. Felock P. Hastings J. Pramanik B. Wolfe A. (1999). Isolation and characterization of novel human immunodeficiency virus integrase inhibitors from fungal metabolites. *Antivir. Chem. Chemother.* 10 63–70. 10.1177/095632029901000202 10335400

[B46] He H. Yang H. Y. Bigelis R. Solum E. H. Greenstein M. Carter G. T. (2002). Pyrrocidines A and B, new antibiotics produced by a filamentous fungus. *Tetrahedron Lett.* 43 1633–1636. 10.1016/S0040-4039(02)00099-0

[B47] Holzapfel C. W. (1968). The isolation and structure of cyclopiazonic acid a toxic metabolite of *Penicillium cyclopium* Westling. *Tetrahedron* 24 2101–2119. 10.1016/0040-4020(68)88113-X 5636916

[B48] Howard B. H. Raistrick H. (1955). Studies in the biochemistry of micro-organisms. 94. The colouring matters of species in the *Aspergillus nidulans* group. I. Asperthecin, a crystalline colouring matter of *Aspergillus quadrilineatus* Thom & Raper. *Biochem. J.* 59 475–484. 14363122 10.1042/bj0590475 PMC1216271

[B49] Jakobi M. Winkelmann G. Kaiser D. Kempler C. Jung G. Berg G. (1996). Maltophilin: a new antifungal compound produced by *Stenotrophomonas maltophilia* R3089. *J. Antibiot.* 49 1101–1104. 10.7164/antibiotics.49.1101 8982338

[B50] Jang J. H. Kanoh K. Adachi K. Shizuri Y. (2006). Awajanomycin, a cytotoxic gamma-lactone-delta-lactam metabolite from marine-derived *Acremonium* sp. AWA16-1. *J. Nat. Prod.* 69 1358–1360. 10.1021/np060170a 16989535

[B51] Jiménez-Osés G. Osuna S. Gao X. Sawaya M. R. Gilson L. Collier S. J. (2014). The role of distant mutations and allosteric regulation on LovD active site dynamics. *Nat. Chem. Biol.* 10 431–436. 10.1038/nchembio.1503 24727900 PMC4028369

[B52] Kato-Noguchi H. Ino T. Ota K. (2008). Secretion of momilactone A from rice roots to the rhizosphere. *J. Plant Physiol.* 165 691–696. 10.1016/j.jplph.2007.07.018 17931745

[B53] Kelley L. A. Mezulis S. Yates C. M. Wass M. N. Sternberg M. J. E. (2015). The Phyre2 web portal for protein modeling, prediction and analysis. *Nat. Protoc.* 10 845–858. 10.1038/nprot.2015.053 25950237 PMC5298202

[B54] Kennedy J. (1999). Modulation of polyketide synthase activity by accessory proteins during lovastatin biosynthesis. *Science* 284 1368–1372. 10.1126/science.284.5418.1368 10334994

[B55] Kettle A. J. Batley J. Benfield A. H. Manners J. M. Kazan K. Gardiner D. M. (2015a). Degradation of the benzoxazolinone class of phytoalexins is important for virulence of *Fusarium pseudograminearum* towards wheat. *Mol. Plant Pathol.* 16 946–962. 10.1111/mpp.12250 25727347 PMC6638480

[B56] Kettle A. J. Carere J. Batley J. Benfield A. H. Manners J. M. Kazan K. (2015b). A γ-lactamase from cereal infecting *Fusarium* spp. catalyses the first step in the degradation of the benzoxazolinone class of phytoalexins. *Fungal Genet. Biol.* 83 1–9. 10.1016/j.fgb.2015.08.005 26296598

[B57] Khaldi N. Seifuddin F. T. Turner G. Haft D. Nierman W. C. Wolfe K. H. (2010). SMURF: genomic mapping of fungal secondary metabolite clusters. *Fungal Genet. Biol.* 47 736–741. 10.1016/J.Fgb.2010.06.003 20554054 PMC2916752

[B58] Kinkel L. L. Schlatter D. C. Bakker M. G. Arenz B. E. (2012). *Streptomyces* competition and co-evolution in relation to plant disease suppression. *Res. Microbiol.* 163 490–499. 10.1016/j.resmic.2012.07.005 22922402

[B59] Kinsella K. Schulthess C. P. Morris T. F. Stuart J. D. (2009). Rapid quantification of *Bacillus subtilis* antibiotics in the rhizosphere. *Soil Biol. Biochem.* 41 374–379. 10.1016/j.soilbio.2008.11.019

[B60] Kokkonen M. Jestoi M. Rizzo A. (2005). The effect of substrate on mycotoxin production of selected *Penicillium* strains. *Int. J. Food Microbiol.* 99 207–214. 10.1016/j.ijfoodmicro.2004.08.014 15734568

[B61] Lewis K. (2013). Platforms for antibiotic discovery. *Nat. Rev. Drug Discov.* 12 371–387. 10.1038/nrd3975 23629505

[B62] Li X. Z. Ma D. Livermore D. M. Nikaido H. (1994). Role of efflux pump(s) in intrinsic resistance of *Pseudomonas aeruginosa*: active efflux as a contributing factor to beta-lactam resistance. *Antimicrob. Agents Chemother.* 38 1742–1752. 10.1128/AAC.38.8.1742 7986004 PMC284631

[B63] Lim F. Y. Hou Y. Chen Y. Oh J. H. Lee I. Bugni T. S. (2012). Genome-based cluster deletion reveals an endocrocin biosynthetic pathway in *Aspergillus fumigatus*. *Appl. Environ. Microbiol.* 78 4117–4125. 10.1128/AEM.07710-11 22492455 PMC3370519

[B64] Livermore D. M. (1998). Beta-lactamase-mediated resistance and opportunities for its control. *J. Antimicrob. Chemother.* 41 25–41. 10.1093/jac/41.suppl_4.25 9688449

[B65] Lynch J. M. Benedetti A. Insam H. Nuti M. P. Smalla K. Torsvik V. (2004). Microbial diversity in soil: ecological theories, the contribution of molecular techniques and the impact of transgenic plants and transgenic microorganisms. *Biol. Fertil. Soils* 40 363–385. 10.1007/s00374-004-0784-9

[B66] Malouin F. Bryan L. E. (1986). Modification of penicillin-binding proteins as mechanisms of beta-lactam resistance. *Antimicrob. Agents Chemother.* 30 1–5. 10.1128/AAC.30.1.1 3530121 PMC176423

[B67] Marschner P. Crowley D. Yang C. H. (2004). Development of specific rhizosphere bacterial communities in relation to plant species, nutrition and soil type. *Plant Soil* 261 199–208. 10.1023/B:PLSO.0000035569.80747.c5

[B68] Materon I. C. Palzkill T. (2001). Identification of residues critical for metallo-beta-lactamase function by codon randomization and selection. *Protein Sci.* 10 2556–2565. 10.1110/ps.40884 11714924 PMC2374027

[B69] Mazzola M. Cook R. J. Thomashow L. S. Weller D. M. Pierson L. S. (1992). Contribution of phenazine antibiotic biosynthesis to the ecological competence of fluorescent pseudomonads in soil habitats. *Appl. Environ. Microbiol.* 58 2616–2624. 1514808 10.1128/aem.58.8.2616-2624.1992 PMC195829

[B70] Mcgrath J. W. Hammerschmidt F. Quinn J. P. (1998). Biodegradation of phosphonomycin by *Rhizobium huakuii* PMY1. *Appl. Environ. Microbiol.* 64 356–358. 9435089 10.1128/aem.64.1.356-358.1998 PMC124718

[B71] Morrissey J. P. Osbourn A. E. (1999). Fungal resistance to plant antibiotics as a mechanism of pathogenesis. *Microbiol. Mol. Biol. Rev.* 63 708–724. 10477313 10.1128/mmbr.63.3.708-724.1999 PMC103751

[B72] Nozawa K. Nakajima S. (1979). Isolation of radicicol from *Penicillium luteo-aurantium*, and meleagrin, a new metabolite, from *Penicillium meleagrinum*. *J. Nat. Prod.* 42 374–377. 10.1021/np50004a004

[B73] O’Driscoll M. Greenhalgh K. Young A. Turos E. Dickey S. Lim D. V. (2008). Studies on the antifungal properties of N-thiolated beta-lactams. *Bioorg. Med. Chem.* 16 7832–7837. 10.1016/j.bmc.2008.06.035 18672374 PMC2617728

[B74] Osbourn A. (2010). Secondary metabolic gene clusters: evolutionary toolkits for chemical innovation. *Trends Genet.* 26 449–457. 10.1016/j.tig.2010.07.001 20739089

[B75] Parkinson A. Klaasen C. D. Watkins J. B. (2001). “Biotransformation of xenobiotics,” in *Casarett & Doull’s Essentials of Toxicology*, eds Klaassen C. D. Watkins J. B. (New York, NY: McGraw-Hill), 133–144. 10.1036/0071470514

[B76] Petersen T. N. Brunak S. von Heijne G. Nielsen H. (2011). SignalP 4.0: discriminating signal peptides from transmembrane regions. *Nat. Methods* 8 785–786. 10.1038/nmeth.1701 21959131

[B77] Reddy P. S. N. Mittapelli V. Reddy V. D. (2010). Antibacterial, antifungal and antifeedant activity of quinazolinonyl-β-lactams/quinazolinones and bis (quinazolinonyl-β-lactams). *Rasayan J. Chem.* 3 635–640.

[B78] Ren H. Liu R. Chen L. Zhu T. Zhu W. M. Gu Q. Q. (2010). Two new hetero-spirocyclic γ-lactam derivatives from marine sediment-derived fungus *Aspergillus sydowi* D2-6. *Arch. Pharm. Res.* 33 499–502. 10.1007/s12272-010-0401-4 20422356

[B79] Riaz K. Elmerich C. Moreira D. Raffoux A. Dessaux Y. Faure D. (2008). A metagenomic analysis of soil bacteria extends the diversity of quorum-quenching lactonases. *Environ. Microbiol.* 10 560–570. 10.1111/j.1462-2920.2007.01475.x 18201196

[B80] Robleto E. A. Borneman J. Triplett E. W. (1998). Effects of bacterial antibiotic production on rhizosphere microbial communities from a culture-independent perspective. *Appl. Environ. Microbiol.* 64 5020–5022. 9835600 10.1128/aem.64.12.5020-5022.1998 PMC90960

[B81] Saunders M. Glenn A. E. Kohn L. M. (2010). Exploring the evolutionary ecology of fungal endophytes in agricultural systems: using functional traits to reveal mechanisms in community processes. *Evol. Appl.* 3 525–537. 10.1111/j.1752-4571.2010.00141.x 25567944 PMC3352505

[B82] Saunders M. Kohn L. M. (2008). Host-synthesized secondary compounds influence the *in vitro* interactions between fungal endophytes of maize. *Appl. Environ. Microbiol.* 74 136–142. 10.1128/AEM.01538-07 17993551 PMC2223208

[B83] Schiff P. L. (2006). Ergot and its alkaloids. *Am. J. Pharm. Educ.* 70:98 10.5688/aj700598 PMC1637017 17149427

[B84] Singh S. B. Li X. Chen T. (2011). Biotransformation of antifungal ilicicolin H. *Tetrahedron Lett.* 52 6190–6191. 10.1016/j.tetlet.2011.09.051

[B85] Soucy S. M. Huang J. Gogarten J. P. (2015). Horizontal gene transfer: building the web of life. *Nat. Rev. Genet.* 16 472–482. 10.1038/nrg3962 26184597

[B86] Stallings J. H. (1954). Soil produced antibiotics—plant disease and insect control. *Bacteriol. Rev.* 18 131–146. 13159784 10.1128/br.18.2.131-146.1954 PMC180792

[B87] Stumpf G. Domdey H. (1996). Dependence of yeast pre-mRNA 3’-end processing on CFT1: a sequence homolog of the mammalian AAUAAA binding factor. *Science* 274 1517–1520. 10.1126/science.274.5292.1517 8929410

[B88] Sun L. H. Dennis B. (2016). *The Superbug that Doctors have been Dreading just Reached the U.S. Washington Post.* Available at: https://www.washingtonpost.com/news/to-your-health/wp/2016/05/26/the-superbug-that-doctors-have-been-dreading-just-reached-the-u-s/?utm_term=.d47b14742fcb [accessed January 1 2017].

[B89] Szewczyk E. Chiang Y. M. Oakley C. E. Davidson A. D. Wang C. C. C. Oakley B. R. (2008). Identification and characterization of the asperthecin gene cluster of *Aspergillus nidulans*. *Appl. Environ. Microbiol.* 74 7607–7612. 10.1128/AEM.01743-08 18978088 PMC2607171

[B90] Taylor D. L. Hollingsworth T. N. McFarland J. W. Lennon N. J. Nusbaum C. Ruess R. W. (2014). A first comprehensive census of fungi in soil reveals both hyperdiversity and fine-scale niche partitioning. *Ecol. Monogr.* 84 3–20. 10.1890/12-1693.1

[B91] Thiele-Bruhn S. (2003). Pharmaceutical antibiotic compounds in soils - A review. *J. Plant Nutr. Soil Sci.* 166 145–167. 10.1002/jpln.200390023

[B92] Thomas P. W. Stone E. M. Costello A. L. Tierney D. L. Fast W. (2005). The quorum-quenching lactonase from *Bacillus thuringiensis* is a metalloprotein. *Biochemistry* 44 7559–7569. 10.1021/bi050050m 15895999

[B93] Thomashow L. S. Bonsall R. F. Weller D. M. (1997). “Antibiotic production by soil and rhizosphere microbes *in situ*,” in *Manual of Environmental Microbiology*, eds Hurst C. J. Knudsen G. R. McInerney M. J. Stetzenbach L. D. Walter M. V. (Washington, DC: ASM Press), 493–499.

[B94] Tipper D. J. (1985). Mode of action of beta-lactam antibiotics. *Pharmacol. Ther.* 27 1–35. 3889939 10.1016/0163-7258(85)90062-2

[B95] VanEtten H. D. Mansfield J. W. Bailey J. A. Farmer E. E. (1994). Two classes of plant antibiotics: phytoalexins versus phytoanticipins. *Plant Cell* 6 1191–1192. 10.1105/tpc.6.9.1191 12244269 PMC160512

[B96] Vatmurge N. S. Hazra B. G. Pore V. S. Shirazi F. Chavan P. S. Deshpande M. V. (2008). Synthesis and antimicrobial activity of beta-lactam-bile acid conjugates linked via triazole. *Bioorg. Med. Chem. Lett.* 18 2043–2047. 10.1016/j.bmcl.2008.01.102 18267360

[B97] Wagenaar M. M. Corwin J. Strobel G. Clardy J. (2000). Three new cytochalasins produced by an endophytic fungus in the genus *Rhinocladiella*. *J. Nat. Prod.* 63 1692–1695. 10.1021/np0002942 11141120

[B98] Walczak P. Pannek J. Boratyński F. Janik-Polanowicz A. Olejniczak T. (2014). Synthesis and fungistatic activity of bicyclic lactones and lactams against *Botrytis cinerea*, *Penicillium citrinum*, and *Aspergillus glaucus*. *J. Agric. Food Chem.* 62 8571–8578. 10.1021/jf502148h 25110806

[B99] Wang J. F. Huang Y. J. Xu Q. Y. Zheng Z. H. Zhao Y. F. Su W. J. (2003). X-ray crystal structure of cytochalasin D produced by *Tubercularia* sp., a novel endophytic fungus of *Taxus mairei*. *J. Chem. Crystallogr.* 33 51–56. 10.1023/A:1021351800842

[B100] Waxman D. J. Strominger J. L. (1983). Penicillin-binding proteins and the mechanism of action of beta-lactam antibiotics. *Annu. Rev. Biochem.* 52 825–869. 10.1146/annurev.bi.52.070183.004141 6351730

[B101] Wei B. Yang Z. D. Chen X. W. Zhou S. Y. Yu H. T. Sun J. Y. (2016). Colletotrilactam A–D, novel lactams from *Colletotrichum gloeosporioides* GT-7, a fungal endophyte of *Uncaria rhynchophylla*. *Fitoterapia* 113 158–163. 10.1016/j.fitote.2016.08.005 27520493

[B102] Weldhagen G. F. (2004). Integrons and beta-lactamases - A novel perspective on resistance. *Int. J. Antimicrob. Agents* 23 556–562. 10.1016/j.ijantimicag.2004.03.007 15194125

[B103] Wheeler T. J. Eddy S. R. (2013). Nhmmer: DNA homology search with profile HMMs. *Bioinformatics* 29 2487–2489. 10.1093/bioinformatics/btt403 23842809 PMC3777106

[B104] Wicklow D. T. Roth S. Deyrup S. T. Gloer J. B. (2005). A protective endophyte of maize: *Acremonium zeae* antibiotics inhibitory to *Aspergillus flavus* and *Fusarium verticillioides*. *Mycol. Res.* 109 610–618. 10.1017/S0953756205002820 16018316

[B105] Wiebe L. A. Bjeldanes L. F. (1981). Fusarin C, a mutagen from *Fusarium moniliforme* grown on corn. *J. Food Sci.* 46 1424–1426. 10.1111/J.1365-2621.1981.Tb04189.X

[B106] Zhang Q. Y. Jones D. M. Saez Nieto J. A. Perez Trallero E. Spratt B. G. (1990). Genetic diversity of penicillin-binding protein 2 genes of penicillin-resistant strains of *Neisseria meningitidis* revealed by fingerprinting of amplified DNA. *Antimicrob. Agents Chemother.* 34 1523–1528. 2121092 10.1128/aac.34.8.1523 PMC171866

[B107] Zhelkovsky A. Tacahashi Y. Nasser T. He X. Sterzer U. Jensen T. H. (2006). The role of the Brr5/Ysh1 C-terminal domain and its homolog Syc1 in mRNA 3’-end processing in *Saccharomyces cerevisiae*. *RNA* 12 435–445. 10.1261/rna.2267606 16431986 PMC1383582

